# Epidemiological Trends and Strain Distribution of Bovine Brucellosis in Vaccinated Industrial Dairy Cattle (2021–2024)

**DOI:** 10.1155/vmi/7432813

**Published:** 2026-05-15

**Authors:** Saeed Alamian, Karim Amiry, Akram Bahreinipour, Maryam Dadar

**Affiliations:** ^1^ Department of Brucellosis, Razi Vaccine and Serum Research Institute (RVSRI), Agricultural Research, Education and Extension Organization (AREEO), Karaj, Iran, areo.ir; ^2^ Health and Management of Zoonotic Diseases, Iranian Veterinary Organization, Tehran, Iran, ivo.org.ir

**Keywords:** *Brucella melitensis*, brucellosis, cattle, epidemiology, Iran, RB51 vaccine, surveillance

## Abstract

Brucellosis remains a significant zoonotic disease in Iran, affecting both public health and livestock productivity despite decades of control efforts. This study presents findings from a four‐year nationwide active surveillance program (2021–2024) targeting industrial dairy cattle herds vaccinated with the *Brucella abortus* RB51 vaccine. The program encompassed 33,994 herds and over 6.2 million blood samples across 32 provinces, representing one of the most comprehensive surveillance initiatives conducted in the country. Serological screening was performed using the Rose Bengal plate test (RBPT), followed by Wright and 2‐ME agglutination tests for confirmation. Lymph node cultures were obtained from 1261 seropositive animals and subjected to classical biotyping and AMOS‐PCR for molecular identification. At the national level, both farm‐ and animal‐level prevalence declined over the study period, highlighting progress in disease management. Farm prevalence peaked in 2022 at 9.7% before decreasing to 6.7% in 2024, while animal prevalence dropped steadily from 0.36% in 2021 to 0.11% in 2024. Similarly, suspected cases declined from 5.8% to 4.2% at the farm level and from 0.16% to 0.07% at the animal level. Provinces such as Isfahan, Tehran, Fars, Yazd, and Qom consistently reported the highest infection burdens, while several regions, including Hormozgan, Mazandaran, Gilan, and Sistan and Baluchestan, showed minimal or no positive detections. Bacteriological culture confirmed *Brucella* infection in 216 animals (17.1%), leading to the isolation of six strains: *B. abortus* biovars 1 and 3, *B. melitensis* biovars 1–3, and the vaccine strain RB51. The predominance of *B. melitensis* biovar 1, particularly in provinces such as Qom and Alborz, underscores the possible risk of cross‐species transmission from small ruminants to cattle. Detection of RB51 in several isolates raises concerns regarding vaccine persistence and its limited protective efficacy against *B. melitensis*. This study demonstrates the persistence of multiple *Brucella* biovars and the circulation of vaccine‐derived strains in vaccinated animals and highlights the need for refined vaccination strategies, stricter biosecurity, and integrated control programs across livestock sectors.

## 1. Introduction

Brucellosis is a zoonotic disease that affects both animals and humans and exhibits considerable regional variation in prevalence. *Brucella*, a genus of Gram‐negative bacteria, causes brucellosis [1]. *Brucella abortus* and *Brucella melitensis* are two predominant species among domestic ruminants in the Middle East [2]. These specified species exhibit high virulence and, alongside domestic animals such as cattle and sheep, cause severe disease in humans. In regions with elevated prevalence, it is occasionally misidentified as febrile diseases such as malaria and typhoid fever. The disease is frequently transmitted to humans through unpasteurized dairy products and exposure to the bodily fluids and secretions of sick animals, particularly in industrial settings. According to recent studies, 1.6–2.1 million people contract brucellosis annually [3]. The majority of these cases are found in high‐risk areas, particularly sub‐Saharan Africa and the Middle East, with most regions of Iran [4, 5]. According to World Health Organization statistics, Iran has approximately 15,000 brucellosis cases each year, second only to Yemen, with over 25,000. Iraq follows as the third highest, with over 10,000 cases each year [6]. Over the past decade, Iran has reported 10–20 per 100,000 cases of human brucellosis in each province [7, 8]. As brucellosis surveillance underreports, true case numbers may be far higher. Cattle brucellosis is known to cause Malta fever. Livestock management and healthy animal reservoirs are needed to control this zoonotic disease in humans [9]. To reduce transmission hazards, vaccination and “test‐and‐slaughter” policies are key interventions [10, 11]. Brucellosis vaccination programs in Iran were launched in 1944. Nonetheless, numerous social, economic, and political issues affected their execution. The immunization effort for cattle in Iran began in 1967 [12]. RB51 is the vaccine used to prevent brucellosis in Iran. Since 2004, brucellosis vaccination for heifers aged 4–6 months has been mandatory, requiring the full dose. Additionally, it is recommended that vaccinated cows have a reduced dosage for revaccination every 2 years [12]. Despite ongoing vaccinations in Iran, brucellosis has increased, and cattle farms have been affected [7, 13, 14]. Although the “test‐and‐slaughter” technique and vaccination initiatives persist, it appears that variables beyond vaccination affect the disease’s prevalence. This study aims to determine the seroprevalence of brucellosis in industrial cattle populations vaccinated against *Brucella abortus* RB51 across different provinces of Iran during 2021–2024, to evaluate the effectiveness of vaccination programs, and identify potential regional variations in disease occurrence.

## 2. Materials and Methods

### 2.1. Ethical Statement

Iranian Veterinary Organization (IVO, Tehran, Iran) approved ethical standards for field study on industrial dairy cattle in June 2022 (Reference: IR. RVSRI.REC1402.002). Animals were slaughtered in accordance with the test‐and‐slaughter protocols established by the IVO.

### 2.2. Study Design and Sampling Framework

From 2021 to 2024, the IVO conducted an active nationwide brucellosis surveillance program to examine all registered industrial dairy farms in Iranian provinces. The study focused on industrial dairy farms registered with the IVO. Blood samples were collected from a representative subset of animals on each farm following standardized protocols. Small farms (≤ 100 cattle) tested all their animals; medium and large farms (101–500 and more cattle) tested 50% of animals across management groups. Provincial veterinary staff regularly collected samples, rotating animals from different management groups to ensure coverage of various types. A total of 6,241,697 blood samples were collected from 33,994 industrial dairy cattle farms, representing 85%–90% of registered farms and 50%–52% of the target industrial cattle population at the time, enabling reliable prevalence estimates (Table S1). A diverse range of specimens was gathered from 32 provinces located in all regions of Iran, including East Azerbaijan, West Azerbaijan, Ardabil, Isfahan, Alborz, Ilam, Bushehr, Tehran, Chaharmahal and Bakhtiari, South Khorasan, Razavi Khorasan, North Khorasan, Kurdistan, Khuzestan, Kerman, Kermanshah, Zanjan, Semnan, Fars, Sistan and Baluchestan, Qazvin, Qom, South Kerman, Kohgiluyeh and Boyer‐Ahmad, Gilan, Golestan, Mazandaran, Lorestan, Hormozgan, Markazi, Hamedan, and Yazd. These locations are vital to the dairy industry and feature dynamic agricultural sectors. The IVO defined industrial dairy farms as facilities housing at least 50 cattle, managed through methods such as confined housing systems, mechanical milking parlors, formulated feeding programs, artificial insemination, regular veterinary oversight, and mandatory participation in national disease surveillance programs. Table S1 illustrates the distribution of animals across different provinces. Herd sizes ranged from 84 to over 10,000. The animals chosen for bacterial culture were selected from routine diagnostic submissions across different regions, allowing the collection of isolates from various geographic areas; however, the selection was not intended to be statistically representative of all nationally reported seropositive animal cases. The national brucellosis control program sent these seropositive animals for slaughter following positive serological results, and lymph node tissue samples were collected from them. Sampling occurred throughout the study period, from 2021 to 2024. Blood samples for serological testing were collected approximately 3 months after RB51 vaccination, in accordance with IVO surveillance protocols. The exact postvaccination interval was not recorded at the individual farm level; only the year and quarter of sampling were documented. For seropositive animals, lymph tissue samples were collected within 2–4 weeks following laboratory confirmation of seropositivity. Provincial veterinary staff were notified of positive results within 1 week of blood collection, and tissue sampling was performed at slaughter. However, due to budgetary constraints, abortion materials were not available for inclusion in this study.

### 2.3. Vaccination Status of Industrial Dairy Cattle Against RB51

The study herds were vaccinated against bovine brucellosis with the RB51 live attenuated *Brucella abortus* vaccine. Calves received a single vaccination between 4 and 8 months of age, in compliance with national brucellosis control protocols. Vaccination in adult cattle was administered solely to nonpregnant animals under veterinary supervision. Pregnant cows were omitted due to the established danger of vaccine‐induced abortion.

All immunizations were administered by certified field veterinarians, and documentation of each animal’s vaccination status, encompassing age, date, and dosage, was preserved in herd health databases. Vaccination was implemented as part of a comprehensive control strategy, alongside regular serological surveillance, test‐and‐slaughter protocols for animals found to be positive, and biosecurity precautions. All industrial farms included in the study follow the recommended revaccination schedule every 2 years. Additionally, animals are vaccinated at a young age with a full dose of the *B. abortus* RB51 vaccine (1.0–3.4 × 10^10^ CFU per animal), administered subcutaneously. Adult animals are also revaccinated with a reduced dose of the vaccine at 12 months of age or older (1.0–3.4 × 10^9^ CFU per animal). The administration method is standardized across farms and remains consistent between young and adult animals. According to IVO rules, blood samples were collected approximately 3 months after RB51 revaccination. The precise interval between vaccination and sampling was not documented; however, since RB51 has no effect on serological diagnosis, this does not influence prevalence estimates for field strains.

### 2.4. Serological Tests

The Rose Bengal plate test (RBPT) was done as the primary diagnostic assay for brucellosis in dairy cattle, as per the IVO’s monitoring program. Animals that pass this screening test will undertake the Wright and 2‐ME agglutination test. IVO recognizes the Wright and 2‐ME test as the standard diagnostic procedure for bovine brucellosis [14]. A positive test indicates infection, whereas a negative test indicates no infection. However, results may sometimes be categorized as “borderline.” In such cases, the examination must be administered up to 3 times, with 3‐week intervals between administrations. After the third test, the veterinarian and IVO’s expertise will decide what to do if the result is uncertain. An animal was classified as seropositive if at least one of the repeated tests produced a clearly positive result, as defined by the predefined cutoff. Animals whose results stayed negative or borderline on all three occasions were classified as seronegative for prevalence calculations.

### 2.5. Lymph Node Culture

Lymph node samples from 1261 seropositive cows at various industrial dairy farms were collected by the IVOs of each province and sent to the Brucellosis Department at the Razi Vaccine and Serum Research Institute for analysis (Karaj, Iran). Lymph node specimens were obtained from the retropharyngeal and supramammary regions of slaughtered animals during control programs, deposited in sterile plastic bags, stored on ice, and promptly transported to the laboratory. The collected samples were kept for future use in isolating *Brucella*. All bacteriological analyses were performed in safety hoods.

Bacterial cultures were performed on lymph node samples collected from seropositive animals. *Brucella* spp. were isolated by inoculating clinical samples onto a Brucella Selective Supplement–containing medium (Oxoid, Basingstoke, UK). This supplement comprised multiple constituents, including cycloheximide (50.00 mg), nystatin (50.00 IU), bacitracin (12.50 IU), polymyxin B (2.50 IU), vancomycin (10.00 mg), and nalidixic acid (2.50 mg). The culture medium utilized consisted of 5.00% inactivated horse serum in *Brucella* agar (HiMedia, Thane, India). The cultures were incubated at 37°C for 14 days in a 10% CO2 atmosphere. Bacterial cultures were removed after 14 days if no growth was seen. Identified colonies of *Brucella* spp. were subsequently transferred to fresh *Brucella* agar for a comprehensive identification and biotyping study.

The culture medium was comprised of trimethoprim (5 mg/mL), rifampin (200 mg/mL), vancomycin (20 mg/mL), cycloheximide (100 mg/mL), bacitracin (25 U/ml), and nystatin (100 U/ml), which were used to identify the RB51 vaccine [15]. As previously mentioned, bacterial phage typing was used to identify the genus and species in situations of bacterial growth [2].

### 2.6. Classical Biotyping

The bacteria were categorized by their ability to produce H2S, CO2 requirement, susceptibility to phage lysis, agglutination by monospecific antisera A and acriflavine, and potential growth at different thionine and basic fuchsin dye concentrations [16]. Tbilisi (Tb) and Izatnagar (IZ) phages were employed to assess strain lysis responses at two dilutions of RTD and RTD × 10^4^. A positive reaction was identified when full lysis transpired after 48 h of incubation at 37°C. This study utilized *B. melitensis* biovar 1 strain 16M, *B. abortus* biovar 1 strain 544, and the vaccine strains RB51 and Rev.1 as control strains. The analysis of the test findings is in accordance with the guidelines specified for bovine brucellosis [14].

### 2.7. Isolation of DNA and AMOS Polymerase Chain Reaction

Following the manufacturer’s instructions, the Favorgen Biotech kit from Taiwan extracted DNA from 215 isolated bacteria. After 24 h at 37°C, bacteria were collected for DNA extraction. DNA integrity was assessed on a 1% agarose gel. The DNA concentration was measured at 260/280 nm using a NanoDrop ND‐1000 (Wilmington, DE, USA). DNA was then stored at −20°C for analysis. Under defined temperature settings, AMOS (*abortus melitensis ovis suis*) polymerase chain reaction was performed on bacterial genomic DNA (Table S2) [17]. Initial denaturation in Step 1 takes 5 min at 95°C. Process 2 requires 30 s of secondary denaturation at 95°C. Step 3 involves 60 s of annealing at 55°C, while Step 4 involves 3 min of 72°C extension. Step 5 ends with a 10‐minute 72°C extension. Steps 2, 3, and 4 were repeated for 35 cycles [17]. The PCR mixture included 12.5 μL of Amplicon Taq PCR Master Mix (South Korea), 0.5 μL of each primer, 7 μL of ddH₂O, and 3 μL of 20 ng/μL template DNA. The study used *B. abortus* biovar 1 strain 544, *B. melitensis* biovar 1 strain 16M, and the vaccine strains Rev.1 and RB51 as positive controls. A separate approach used sterile water as the negative control instead of DNA. PCR findings were analyzed on 1% agarose gel.

### 2.8. Statistical Analysis

The provincial and annual animal‐level prevalence was calculated by dividing the number of seropositive animals by the total number of animals tested and then converting the result to a percentage. Using the same approach, farm‐level prevalence was calculated as the number of farms with at least one positive animal divided by the total number tested. Wilson’s score 95% CIs were calculated for all prevalence values to assess accuracy.

The Cochran–Armitage trend test was used to examine secular trends in animal‐ and farm‐level prevalence over 4 years. It tests if the percentage of positives changes steadily. The national aggregate statistics and those for each province were tested separately when the sample sizes were large enough. The prevalence difference between provinces was examined using the chi‐square test of homogeneity. To quantify interprovincial differences, binomial logistic regression models used province and year as categorical independent variables. Using odds ratios (ORs) with 95% CI, we compared the province risk to a common reference.

West Azerbaijan was chosen as the reference category because of its low but nonzero livestock and farm frequency during the study. This choice provided a steady baseline and avoided issues with zero‐event province estimation. To analyze the independent impacts of year and province while considering the binomial outcome, two binomial logistic regression models were created: positive animals out of the total animals tested in each province‐year strata and the number of positive farms out of all tested farms. Both models included category fixed effects (2021 base year, 2022, 2023, and 2024 indicator variables) and province as a fixed effect, with West Azerbaijan as the reference. Spearman’s rank correlation coefficient was used to assess associations between cumulative animal prevalence and strain characteristics (diversity and proportion of *B. melitensis*) for 20 provinces with culture‐positive isolates.

## 3. Result

**FIGURE 1:**
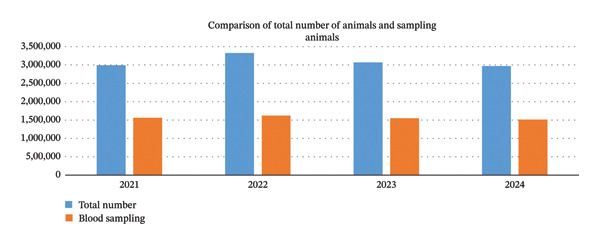
Comparison of total number of animals and blood sampling (2021–2024).

Based on the seroprevalence study, the total animal population of industrial dairy cattle peaked in 2022 at about 3.3 million and then gradually declined in subsequent years. Blood sampling also reached its highest level in 2022, at roughly 1.65 million, and consistently accounted for about half of the total population of industrial dairy cattle throughout the entire study period. Overall, the sampling rate remained steady, fluctuating between 50% and 52% of the total population over the 4‐year period (Figure 1, Table S1).

Between 2021 and 2024, a significant amount of blood samples was obtained each year, with figures fluctuating between 1.51 million and 1.62 million. The peak number of samples occurred in 2022 (1,620,096), followed by a gradual decrease in subsequent years. The number of sampled farms peaked in 2023 at 8968 but declined slightly in 2024 to 8428. The number of positive samples (farms) varied annually, reaching a peak of 866 in 2022 and subsequently declining steadily to 565 by 2024. Correspondingly, the positive animal samples showed a significant decline, from 5572 in 2021 to merely 1731 in 2024. Regarding suspected cases, both farms and animals exhibited a declining tendency (Table 1, Tables 2 and 3).

**TABLE 1:** National summary of brucellosis active surveillance in cattle, Iran (2021–2024).

Year	Farms tested (*N*)	Animals tested (*N*)	Positive farms (*n*)	Positive animals (*n*)	Farm‐level prevalence % (95% CI)	Animal‐level prevalence % (95% CI)
2021	7675	1,562,706	683	5572	8.90 (8.26–9.56)	0.36 (0.35–0.37)
2022	8923	1,620,096	866	3531	9.71 (9.10–10.34)	0.22 (0.21–0.23)
2023	8968	1,546,135	747	2455	8.33 (7.76–8.92)	0.16 (0.15–0.17)
2024	8428	1,512,760	565	1731	6.70 (6.18–7.26)	0.11 (0.11–0.12)
Total	33,994	6,241,697	2861	13,289	—	

*Note:* CI: confidence interval calculated using the Wilson score method. Cochran–Armitage test for trend in animal‐level prevalence: *Z* = −45.3, *p* < 0.001. Cochran–Armitage test for trend in farm‐level prevalence: *Z* = −8.1, *p* < 0.001.

**TABLE 2:** Province‐level animal brucellosis prevalence and temporal trends in Iran (2021–2024).

Province	Total animals tested	Total positive animals	Prevalence % (95% CI)	Trend (2021–2024)
*High prevalence (> 0.5%)*				
Lorestan	51,291	287	0.56 (0.50–0.63)	Decreasing
Qom	133,328	941	0.71 (0.66–0.75)	Decreasing
Yazd	85,286	1163	1.36 (1.29–1.44)	Decreasing
South Kerman^∗^	863	162	18.77 (16.25–21.50)	Outbreak

*Medium prevalence (0.1%–0.5%)*				
Alborz	460,055	661	0.14 (0.13–0.16)	Stable
Bushehr	2615	5	0.19 (0.08–0.45)	Stable
Chaharmahal and Bakhtiari	138,316	530	0.38 (0.35–0.42)	Increasing
East Azerbaijan	124,597	162	0.13 (0.11–0.15)	Increasing
Fars	282,101	1207	0.43 (0.40–0.45)	Decreasing
Hamedan	112,857	341	0.30 (0.27–0.34)	Increasing
Isfahan	1,082,037	1854	0.17 (0.16–0.18)	Decreasing
Kerman	128,425	202	0.16 (0.14–0.18)	Stable
Kohgiluyeh and Boyer‐Ahmad	27,478	39	0.14 (0.10–0.19)	Increasing
Semnan	69,447	124	0.18 (0.15–0.21)	Decreasing
South Khorasan	55,833	88	0.16 (0.13–0.19)	Decreasing
Tehran	1,719,258	4564	0.27 (0.26–0.27)	Decreasing

*Low prevalence (< 0.1%)*				
Ardabil	100,929	30	0.03 (0.02–0.04)	Decreasing
Gilan	8611	2	0.02 (0.00–0.08)	Stable
Golestan	45,378	38	0.08 (0.06–0.11)	Increasing
Hormozgan	867	0	0.00 (0.00–0.42)	No cases
Ilam	14,051	6	0.04 (0.02–0.09)	Stable
Kermanshah	130,683	130	0.10 (0.08–0.12)	Decreasing
Razavi Khorasan	344,358	298	0.09 (0.08–0.10)	Decreasing
Khuzestan	26,490	15	0.06 (0.03–0.10)	Increasing
Kurdistan	56,653	31	0.05 (0.04–0.08)	Stable
Markazi	180,275	114	0.06 (0.05–0.08)	Unclear
Mazandaran	45,886	0	0.00 (0.00–0.01)	No cases
North Khorasan	29,837	7	0.02 (0.01–0.05)	Stable
Qazvin	682,630	260	0.04 (0.03–0.04)	Stable
Sistan and Baluchestan	9414	3	0.03 (0.01–0.09)	Decreasing
West Azerbaijan	52,897	13	0.02 (0.01–0.04)	Stable
Zanjan	47,466	12	0.03 (0.01–0.04)	Stable

*Note:* CI: confidence interval calculated using the Wilson score method for binomial proportions. Trend was qualitatively assessed from year‐by‐year prevalence and confirmed by logistic regression with year as a continuous variable (*p* for trend).

^∗^South Kerman experienced a focal outbreak in 2022 (161 positives out of 477 tested) and is presented separately.

**TABLE 3:** Province‐level farm brucellosis prevalence and temporal trends in Iran, 2021–2024.

Province	Total farms tested	Total positive farms	Farm prevalence % (95% CI)	Trend (2021–2024)
*High prevalence (> 10%)*				
Ardabil	201	26	12.94 (8.13–16.77)	Decreasing
Isfahan	2430	456	18.77 (17.22–20.41)	Decreasing
Qom	1923	318	16.54 (14.95–18.25)	Decreasing
Yazd	1295	290	22.39 (20.18–24.75)	Decreasing
Tehran	4070	480	11.79 (10.83–12.82)	Decreasing
Alborz	1242	195	15.70 (13.76–17.83)	Stable

*Medium prevalence (5%–10%)*				
Bushehr	100	5	5.00 (1.98–11.68)	Stable
Chaharmahal and Bakhtiari	2267	200	8.82 (7.70–10.08)	Increasing
Fars	2528	240	9.49 (8.40–10.71)	Decreasing
Hamedan	689	45	6.53 (4.87–8.66)	Increasing
Kohgiluyeh and Boyer‐Ahmad	296	16	5.41 (3.29–8.70)	Stable
Qazvin	1375	81	5.89 (4.74–7.29)	Decreasing
Semnan	1131	58	5.13 (3.97–6.58)	Decreasing

*Low prevalence (< 5%)*				
East Azerbaijan	1059	32	3.02 (2.15–4.24)	Increasing
Gilan	191	2	1.05 (0.18–4.11)	Stable
Golestan	758	27	3.56 (2.44–5.16)	Increasing
Hormozgan	6	0	0.00 (0.00–39.03)	No cases
Ilam	380	5	1.32 (0.51–3.19)	Stable
Kerman	2293	80	3.49 (2.80–4.33)	Stable
Kermanshah	638	17	2.66 (1.63–4.27)	Decreasing
Razavi Khorasan	2767	115	4.16 (3.46–4.98)	Decreasing
Khuzestan	268	5	1.87 (0.71–4.52)	Increasing
Kurdistan	631	10	1.58 (0.82–2.96)	Stable
Lorestan	1277	46	3.60 (2.69–4.79)	Decreasing
Markazi	455	20	4.40 (2.82–6.76)	Unclear
Mazandaran	420	0	0.00 (0.00–0.91)	No cases
North Khorasan	429	7	1.63 (0.73–3.45)	Stable
South Khorasan	1505	60	3.99 (3.09–5.12)	Decreasing
Sistan and Baluchestan	50	1	2.00 (0.10–11.99)	Decreasing
West Azerbaijan	860	7	0.81 (0.37–1.73)	Stable
Zanjan	428	7	1.64 (0.74–3.45)	Stable

*Outbreak*				
South Kerman	32	10	31.25 (17.30–49.22)	Outbreak

*Note:* CI: confidence interval calculated using the Wilson score method for binomial proportions. Trend was qualitatively assessed from year‐by‐year farm prevalence. South Kerman experienced a focal outbreak in 2022 (9/9 farms positive, 100%) and is presented separately.

The number of suspected farms increased to 631 in 2022 but plummeted to 355 in 2024, while the count of suspected animals steadily declined from 2456 in 2021 to 1072 in 2024. Despite constant sampling, both positive detections and suspected cases showed a persistent decline over the four years, indicating improved disease control. Isfahan, Tehran, Fars, Yazd, and Qom consistently had the highest number of positive cases, with Isfahan alone reporting 141 farms (754 animals) in 2021. Chaharmahal, Bakhtiari, Ardabil, and Razavi Khorasan had moderate infection rates, while Hormozgan, Gilan, Mazandaran, and Sistan and Baluchestan had few or no cases. Despite geographical disparities, both positive and suspected cases declined after 2022, suggesting that vaccination and control efforts may have reduced the prevalence of industrial cattle brucellosis.

The national animal‐level prevalence showed a statistically significant decreasing trend from 0.36% in 2021 to 0.11% in 2024 (*p* < 0.001). Farm‐level prevalence also decreased significantly, particularly after a 2022 peak. The confidence intervals are narrow due to the large sample size, indicating high precision in these national estimates.

The prevalence at the farm level is significantly higher (6.70%–9.71%) than that at the vaccinated‐animal level (0.11%–0.36%). The prevalence of both farms and animals reaches its highest in 2022 and subsequently diminishes consistently until 2024 (Table 2).

The likelihood of seropositivity at the animal level decreased significantly each year compared to 2021 (OR = 0.30, 95% CI: 0.28–0.32). A similar declining trend was observed at the farm level, although it was less evident in 2022 (OR = 0.96, 95% CI: 0.86–1.07), becoming clearer in 2023 and 2024 (Table 4). After adjusting for the year, there was notable variation in risk. Compared to West Azerbaijan, provinces like Qom, Yazd, Lorestan, and Fars had much higher odds of being positive at both levels. The high OR in South Kerman indicates the main outbreak in 2022. These findings demonstrate that both year and province are strong independent predictors of *Brucella* prevalence. They help identify where interventions should be targeted and assess the effectiveness of control efforts over time.

**TABLE 4:** Association of year and province with *Brucella* seropositivity in cattle (animal level) and farm positivity (farm level), Iran, 2021–2024.

Variable	Animal‐level model OR (95% CI)	Farm‐level model OR (95% CI)
*Year (ref = 2021)*		
2022	0.60 (0.57–0.63)	0.96 (0.86–1.07)
2023	0.43 (0.41–0.46)	0.79 (0.71–0.89)
2024	0.30 (0.28–0.32)	0.61 (0.54–0.69)

*Province (ref = West Azerbaijan)*		
Alborz	7.01 (4.01–12.27)	24.24 (11.32–51.89)
Ardabil	1.46 (0.75–2.85)	19.68 (8.56–45.26)
Bushehr	9.50 (3.34–27.03)	6.67 (2.07–21.49)
Chaharmahal and Bakhtiari	19.04 (10.92–33.21)	12.27 (5.70–26.42)
East Azerbaijan	6.48 (3.67–11.44)	3.92 (1.72–8.94)
Fars	21.32 (12.28–37.02)	12.91 (6.04–27.59)
Gilan	1.15 (0.26–5.12)	1.32 (0.27–6.48)
Golestan	4.18 (2.19–7.98)	4.58 (1.97–10.68)
Hamedan	14.99 (8.57–26.22)	8.50 (3.84–18.83)
Hormozgan	—^∗^	—^∗^
Ilam	2.12 (0.80–5.59)	1.70 (0.54–5.40)
Isfahan	8.49 (4.91–14.70)	28.84 (13.54–61.44)
Kerman	7.96 (4.53–13.98)	4.71 (2.16–10.29)
Kermanshah	4.94 (2.77–8.82)	3.35 (1.45–7.73)
Razavi Khorasan	4.30 (2.45–7.54)	5.23 (2.42–11.30)
Khuzestan	2.81 (1.33–5.93)	2.34 (0.87–6.26)
Kohgiluyeh and Boyer‐Ahmad	7.02 (3.58–13.78)	6.80 (2.73–16.95)
Kurdistan	2.73 (1.42–5.25)	2.02 (0.76–5.33)
Lorestan	27.79 (15.83–48.77)	4.73 (2.11–10.59)
Markazi	3.15 (1.77–5.60)	5.70 (2.56–12.68)
Mazandaran	—^∗^	—^∗^
North Khorasan	1.31 (0.52–3.30)	2.04 (0.77–5.41)
Qazvin	1.89 (1.06–3.38)	7.65 (3.49–16.74)
Qom	35.06 (20.18–60.91)	24.03 (11.37–50.78)
Semnan	8.86 (4.97–15.80)	6.73 (3.05–14.85)
Sistan and Baluchestan	1.58 (0.45–5.60)	2.54 (0.31–20.91)
South Khorasan	7.89 (4.42–14.09)	5.16 (2.31–11.53)
South Kerman	938.23 (500.80–1758.07)	57.51 (20.53–161.14)
Tehran	13.16 (7.60–22.77)	16.45 (7.74–34.96)
Yazd	66.90 (38.33–116.80)	38.53 (18.00–82.48)
Zanjan	1.26 (0.57–2.77)	2.07 (0.77–5.57)

*Note:* OR: the odds ratio after accounting for every variable in the table. CI stands for 95% confidence interval. To prevent estimation issues, Hormozgan and Mazandaran were removed from the animal and farm level models, respectively, because they had no positive animals or farms. The number of positive animals among all animals tested in each province and year was used in the animal‐level model. The number of farms that tested positive was used in the farm‐level model. Binomial logistic regression, a generalized linear model with a logit link, was used to fit each model. Due to its consistently low, nonzero prevalence in both datasets, West Azerbaijan was selected as the reference province. The (—^∗^) indicate provinces excluded from that specific model due to zero positive cases.

**FIGURE 2:**
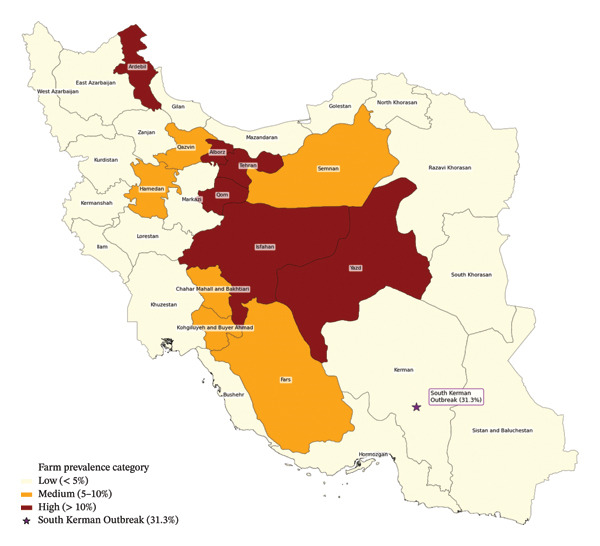
Trends in farm prevalence in all provinces of Iran with positive cases (2021–2024).

The frequency on farms peaked at 9.7% in 2022, thereafter declining to 6.7% by 2024. The prevalence of animals declined steadily over 4 years, from 0.36% in 2021 to 0.11% in 2024. Likewise, suspected farms increased marginally in 2022 (7.1%) before diminishing to 4.2% by 2024, whereas suspected animals consistently declined from 0.16% in 2021 to merely 0.07% in 2024 (Figure 2, Tables 2 and 3).

Farm‐level prevalence increased slightly from 8.9% in 2021 to a peak of 9.7% in 2022 and then declined steadily to 6.7% in 2024. The animal‐level prevalence decreased steadily from 0.36% in 2021 to 0.11% in 2024, with the highest value observed in 2021 (Table 1). During the 4‐year active surveillance program (2021–2024), 1261 cow samples were collected from vaccinated industrial dairy farms across several Iranian provinces.

**FIGURE 3:**
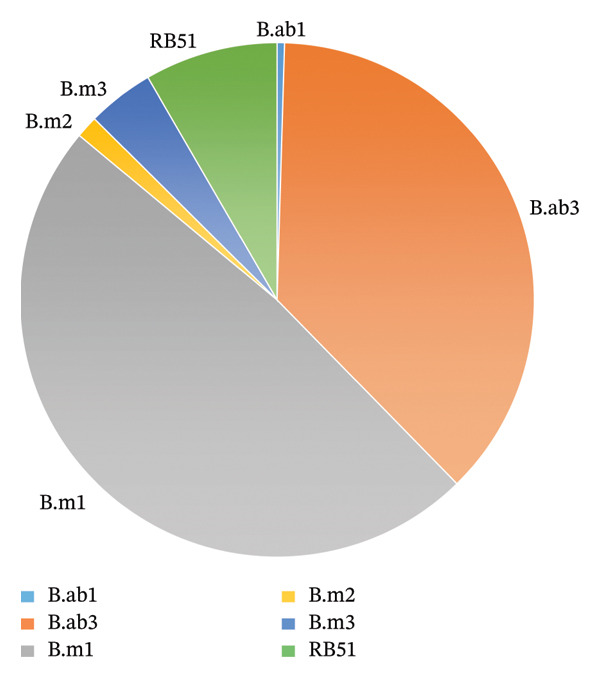
Isolation of *Brucella* strains from vaccinated cattle under active surveillance in Iran (2021–2024).

Among these, 216 samples (17.1%) proved positive in culture, resulting in the isolation of six *Brucella* strains: *B. abortus* biovar 1 (B.ab1), *B. abortus* biovar 3 (B.ab3), *B. melitensis* biovar 1 (B.m1), *B. melitensis* biovar 2 (B.m2), *B. melitensis* biovar 3 (B.m3), and the vaccine strain RB51. Qom (62/131, 47.3%), Alborz (23/51, 45.1%), Kermanshah (17/45, 37.8%), Lorestan (17/53, 32.1%), and Semnan (12/40, 30%) had the highest proportions of culture‐positive samples. In particular, B.m1 was the most commonly isolated strain, followed by RB51 and *B. abortus* biovar 3. B.m2, B.m3, and *B. abortus* biovar 1 were less common but were found in numerous provinces. Ilam, Bushehr, South Khorasan, North Khorasan, Khuzestan, Sistan and Baluchestan, Kurdistan, South Kerman, Gilan, Mazandaran, and Hormozgan had no positive isolates during surveillance (Figure 3, Table 5). Among the 216 isolates, 18 (8.3%) were identified as the RB51 vaccine strain. Some of the cattle that received the reduced vaccine dose still had RB51 in their lymph nodes three months later. *B. abortus* or a mix of *B. abortus* and RB51 is more common in provinces with high prevalence (Qom, Yazd, and Lorestan), while *B. melitensis* biovars are more prevalent in provinces with lower prevalence. RB51 has been identified in 10 provinces, often with field strains. AMOS‐PCR successfully detected *B. melitensis* with a specific PCR product of 731 bp, consistent with the identification of wild‐type *B. melitensis*. In addition, AMOS‐PCR identified the *B. abortus*–specific band (498 bp) exclusively in biovar 1 and 2 isolates, confirming its capability to differentiate *B. abortus* biovars 1, 2, and 4. Supplementary primers were employed to differentiate wild‐type *B. abortus* from vaccine strains of RB51 used on cattle farms (Table S2). In this reaction, the positive control amplified a 400 bp fragment from the field *B. abortus* and 900‐ to 1300‐base‐pair fragments from the RB51 DNA segment associated with the vaccine strain.

**TABLE 5:** Relationship between animal‐level brucellosis prevalence and circulating *Brucella* strains in Iranian provinces, 2021–2024.

Province	Animal prevalence % (95% CI)	Total culture positives	Dominant strain(s)	Strain diversity (no. of types)	*B. melitensis* proportion	*B. abortus* proportion	RB51 isolated
*High prevalence (> 0.5%)*							
Lorestan	0.56 (0.50–0.63)	17	B.m1	2	0.94	0.00	Yes
Qom	0.71 (0.66–0.75)	62	B.ab3	4	0.06	0.92	Yes
Yazd	1.36 (1.29–1.44)	4	B.ab3/RB51	2	0.00	0.50	Yes

*Medium prevalence (0.1%–0.5%)*							
Alborz	0.14 (0.13–0.16)	23	B.m1	2	0.83	0.00	Yes
Chaharmahal and Bakhtiari	0.38 (0.35–0.42)	5	B.m1	2	0.60	0.00	Yes
East Azerbaijan	0.13 (0.11–0.15)	2	B.m1	1	1.00	0.00	No
Fars	0.43 (0.40–0.45)	7	B.m1	3	0.43	0.29	Yes
Hamedan	0.30 (0.27–0.34)	5	B.ab3/B.m1	3	0.40	0.40	Yes
Isfahan	0.17 (0.16–0.18)	15	B.ab3	3	0.33	0.67	No
Kerman	0.16 (0.14–0.18)	11	B.m1	3	1.00	0.00	No
Kermanshah	0.10 (0.08–0.12)	17	B.m1	2	0.88	0.00	Yes
Kohgiluyeh and Boyer‐Ahmad	0.14 (0.10–0.19)	2	B.m1	1	1.00	0.00	No
Semnan	0.18 (0.15–0.21)	12	B.m1	2	0.83	0.00	Yes
Tehran	0.27 (0.26–0.27)	13	B.ab3	2	0.31	0.69	No

*Low prevalence (< 0.1%)*							
Ardabil	0.03 (0.02–0.04)	4	B.m3	1	1.00	0.00	No
Golestan	0.08 (0.06–0.11)	1	B.m1	1	1.00	0.00	No
Markazi	0.06 (0.05–0.08)	4	B.m1	1	1.00	0.00	No
Qazvin	0.04 (0.03–0.04)	8	B.m1	2	0.88	0.00	Yes
West Azerbaijan	0.02 (0.01–0.04)	3	B.m1	1	1.00	0.00	No
Zanjan	0.03 (0.01–0.04)	1	B.m2	1	1.00	0.00	No

*Outbreak*							
South Kerman^∗^	18.77 (16.25–21.50)	0	—	—	—	—	—

^∗^South Kerman had a focal outbreak in 2022, but no culture isolates were obtained.

To assess the association between strain characteristics and prevalence, Spearman rank correlation coefficients were calculated for the 20 provinces with culture‐positive isolates (Table 6). It showed that there is no clear link between high prevalence and high strain diversity.

**TABLE 6:** Correlation analysis between prevalence and diversity.

Comparison	Spearman’s *ρ*	*p* value	Interpretation
Prevalence vs. strain diversity	0.52	0.02	Moderate positive correlation—provinces with higher prevalence tend to harbor a greater variety of *Brucella* strains.
Prevalence vs. proportion of *B. melitensis*	−0.78	< 0.001	Strong negative correlation—provinces with higher prevalence have a lower proportion of *B. melitensis*, indicating dominance of *B. abortus* in high‐burden areas.

## 4. Discussion

This study aimed to determine the prevalence of brucellosis among industrially vaccinated dairy cattle and on farms. The seroprevalence findings from 2021 to 2024 indicate significant trends in sampling methodologies and disease dynamics. The peak in total animal population, recorded in 2022 (about 3.3 million), coincided with the highest number of blood samples obtained (around 1.65 million), with a stable sampling rate of 50%–52%. This illustrates a comprehensive surveillance technique throughout the study duration in vaccinated animals. Furthermore, the highest number of farms sampled was recorded in 2023 (8968), demonstrating extensive experience in expanding the geographic scope of monitoring across all regions of industrial dairy farms. Upon comparing our data with prior seroprevalence studies of infectious diseases in livestock, such as brucellosis across various locations, we identify both similarities and discrepancies. This is the first study to report the seroprevalence of brucellosis on RB51‐vaccinated dairy cattle farms at both the individual and herd levels.

Prior meta‐analyses and surveillance data demonstrate that brucellosis seroprevalence exhibits annual fluctuations while also indicating that control strategies have effectively diminished prevalence over time [7, 18]. A meta‐analysis of brucellosis studies from 2019 to 2024 indicated a greater seroprevalence in the initial years (about 5.5% in 2019–2021) and a decrease to roughly 3.9% in 2022–2024, signifying enhanced national control measures. A national surveillance study in China revealed variable yet generally increasing or decreasing patterns in bovine brucellosis seroprevalence, contingent upon location and time, underscoring the necessity of regional control strategies [19]. The current findings emphasize the need to maintain strict monitoring and control measures to ensure continued reductions in illness rates. The study also shows that Isfahan, Tehran, Fars, Yazd, and Qom provinces consistently reported the highest incidence of brucellosis in industrial cow farms, supporting recent Iranian research [20]. A 2025 epidemiological study across 32 provinces in 2023 indicated elevated seroprevalence rates at the farm level in Qom (22.8%), Isfahan (18.1%), and Yazd (14.7%), as well as in Alborz and Tehran provinces. Provinces such as Chaharmahal and Bakhtiari, Ardabil, and Razavi Khorasan, identified as exhibiting moderate infection rates, have previously shown a variable but moderate incidence of brucellosis cases, which aligns with these findings [20]. The provinces of Hormozgan, Gilan, Mazandaran, and Sistan and Baluchestan, which exhibit few or no cases here, are also regularly described as having low seroprevalence or brucellosis incidence in brucellosis studies across Iran, corroborating this geographical disparity trend [13]. The decrease in positive and suspected brucellosis cases after 2022 is consistent with other Iranian studies that show vaccination and control strategies, such as test‐and‐slaughter programs, reduce industrial cattle brucellosis. Although vaccination coverage gaps and vaccine efficacy vary, especially for the RB51 vaccine used widely in Iran, continuous control measures have contributed to this drop, according to research [12].

Our study found that farm‐level brucellosis prevalence (≃6%–10%) exceeded vaccinated‐animal prevalence (≃0.1%–0.4%), peaking in 2022 and then slowly declining through 2024. Recent data on Iran’s industrial dairy sector support this pattern. For example, in an epidemiological study of bovine brucellosis in Iran, farm‐level prevalence averaged 7.6%, with rates as high as 22.8% in some areas, such as Qom, and animal‐level prevalence averaged 0.4% in industrial dairy cattle [20]. Because farms are considered positive if at least one animal tests positive, farm prevalence aggregates disease presence, while animal prevalence shows the proportion of animals infected. The high prevalence in 2022 and the subsequent decline are compatible with expanded vaccination and control efforts that improved biosecurity on Iranian dairy farms. Similar Iranian research has shown that herd management, hygiene, and mobility controls reduce brucellosis. A case–control study found that farm‐level risk factors, including new animals and poor cleanliness, increased infection risk, whereas excellent disinfection methods decreased it, although the study reports that proper RB51 vaccination (with full or reduced doses) shows no protective effect against brucellosis [21]. Furthermore, another study reported a high prevalence of brucellosis in cattle with a history of RB51 vaccination [22].

RB51‐vaccinated industrial dairy cattle in Iran have been monitored in this study for 4 years, revealing *Brucella* persistence and diversity across vaccinated farms. From 1261 samples collected between 2021 and 2024, 216 (17.1%) were culture‐positive, yielding six *Brucella* strains, with *B. melitensis* biovar 1 dominating, followed by RB51 and *B. abortus* biovar 3, highlighting the diverse epidemiology of vaccinated herds.

The highest prevalence of culture‐positive cases was in Qom (47.3%), Alborz (45.1%), and Kermanshah (37.8%), while Ilam, Bushehr, and Sistan and Baluchestan had no positive isolates, suggesting spatial heterogeneity in infection dynamics and possible differences in management practices, biosecurity, or herd immunity. *B. abortus* may persist or be transmitted within bovine populations, whereas *B. melitensis*, likely originating from small ruminants, is more common in low‐prevalence areas. Long‐standing endemicity or numerous introductions may explain strain diversity in high‐prevalence provinces. Qom has many *Brucella* species, while Yazd has the highest prevalence but the least diversity. This indicates that management practices and animal movement, rather than strain diversity alone, are principal determinants of prevalence.

RB51 protects 70%–87% of cattle from experimental infections, depending on area and management. Protection may be shorter than 4 years, necessitating boosters; however, their necessity is debatable [23]. The isolation of the RB51 vaccine strain from 18 animals across 10 provinces (Alborz, Chaharmahal and Bakhtiari, Fars, Hamedan, Kermanshah, Lorestan, Qazvin, Qom, Semnan, and Yazd) was regarded as a significant discovery, representing 8.3% of all culture‐positive isolates. In Spain, in one mass‐vaccination program, RB51 was cultured from 78 out of 897 declared abortions (∼8.7%) following vaccination of ∼14,900 pregnant cows [24]. The isolation of RB51 from clinical samples in this study (8.3%) is consistent with the documented capacity of this vaccine strain to persist in vaccinated animals in Spain and, in rare instances, cause disease in both animals and humans, as first reported in Chile [25].

The inclusion of *B. abortus* and *B. melitensis* strains in the same surveillance program of industrial dairy cattle farms underlines the difficulty of cross‐species transmission. Other studies have isolated *Brucella* biovars, including vaccine strain RB51, from vaccinated herds [26–28]. In Iran, Spain, and Chile, RB51 was discovered in vaccinated herds with abortion epidemics, raising concerns regarding virulence and excretion [27, 29]. Field strains often coexist with vaccination strains in endemic areas. The high incidence of *B. melitensis* isolates in Iranian RB51‐vaccinated dairy cattle suggests that vaccination does not protect against infection. RB51 is effective against *B. abortus* but not *B. melitensis*, according to various investigations [27, 30]. *B. melitensis* was also isolated from cattle in 12 provinces, comprising 71.5% of all isolates. Given that small ruminants are the primary natural reservoirs of this species [16, 28], this pattern may reflect spillover from sheep and goat populations, particularly in areas where mixed farming is common.

Furthermore, consistent with our results, a recent Iranian study found that *B. melitensis* biovar 1 was the most prevalent strain recovered from vaccinated cattle, demonstrating the vaccine’s inability to prevent dairy herd infection or shedding [31, 32]. The RB51 vaccine, licensed for *B. abortus* control, has limitations globally for *B. melitensis*. It has not eliminated brucellosis in endemic locations where both species circulate, and sheep/goat–cattle cross‐infection complicates control. The vaccine does not elicit antibodies detectable by traditional assays, making diagnosis difficult [12, 27]. Thus, the Iranian data replicate global observations; the widespread isolation of *B. melitensis* from RB51‐vaccinated cattle confirms that RB51 does not provide cross‐protection against *B. melitensis*. The primary advantage of RB51 is its DIVA (Differentiating Infected from Vaccinated Animals) capability, not efficacy against heterologous species. Combining RB51 with *B. melitensis* Rev.1 would negate this DIVA advantage and is not recommended. Therefore, improved vaccines that protect against both *B. abortus* and *B. melitensis* while retaining DIVA properties are urgently needed.

There may be several confounders that are not included in the national surveillance database that could affect the observed prevalence patterns. Some of these include animal movements between provinces, herd size, farming with different species (cattle with small ruminants), small‐ruminant density in a given area, cattle imports, differences in provincial surveillance intensity, biosecurity practices, and diagnostic frequency. For instance, the high prevalence in Qom and Yazd may be partly due to the many animal trade networks in those areas rather than to local epidemiological conditions alone. On the other hand, the low prevalence in provinces like West Azerbaijan may be due to stronger biosecurity measures or geographic isolation from the national reference laboratory. While these unmeasured factors constrain causal inference, the persistent decline across most provinces corroborates the efficacy of national control initiatives. Subsequent research integrating these variables is essential to enhance risk assessment and accurately guide interventions.

In this study, we hypothesize that *B. melitensis* infection in cattle may originate from spillover events in small ruminant reservoirs, given the species’ established host preference and analogous observations in other countries. However, we lacked the necessary phylogenetic data (e.g., MLVA and whole genome sequencing) and livestock network information (e.g., movement patterns and mixed farming density) to conclusively demonstrate transmission pathways.

Furthermore, the results of this study should be viewed with caution due to its observational surveillance design. It did not include an unvaccinated control group, a matched‐cohort comparison, or adjustments for potential confounders, so it was not intended to evaluate the RB51 vaccine’s effectiveness. However, a 3‐month postvaccination sampling interval is insufficient to assess vaccine effectiveness, and the observed decrease in prevalence may be due to other factors. Therefore, the findings are better considered as epidemiological observations rather than direct evidence of vaccine efficacy. The continued presence of brucellosis in the study area highlights the complexity of disease ecology in endemic multihost systems, where factors such as animal movement, cross‐species transmission, herd management, and variations in biosecurity can sustain disease transmission. These observations underscore the challenge of controlling brucellosis with a single intervention and emphasize the importance of integrated strategies that encompass vaccination, surveillance, movement controls, and biosecurity. Additionally, we clarify that the molecular results should be viewed as indicative of the circulating strains detected within the cultured subset, rather than a definitive representation of the nationwide strain distribution.

## 5. Conclusion

The reported decline in seroprevalence specifically referred to the overall serological results observed in the cattle population during the surveillance period and did not indicate complete protection against *B. melitensis*. The isolation of *B. melitensis* from lymph nodes suggested that cross‐species transmission likely occurred in some areas where cattle were epidemiologically linked to nearby small ruminant populations. Although sheep and goats are not frequently kept on industrial dairy farms, it is still possible for *Brucella* spp. to spread between species. In many parts of Iran, small ruminant flocks are raised on nearby industrial farms and may share veterinary services and management networks with cattle farms. Such indirect contact can facilitate the spread of *Brucella* spp. between species. Animal movement, contaminated equipment, personnel, or environmental exposure may also facilitate the transmission of the pathogen among adjacent livestock populations.

This work gives valuable insights into the epidemiology and prevalence of brucellosis in vaccinated Iranian dairy cattle under active surveillance, although it has several limitations.

First, the surveillance included serological tests in IVO programs, such as RBPT, SAT, and 2‐ME, and culture, which are standard but have limited sensitivity and specificity, as a significant portion of infected animals might be undetected using RBPT as a screening test [33]. Due to *Brucella*’s diligence and the possibility of false negatives, culture methods may underestimate prevalence, especially in chronically infected or intermittently shedding animals. Second, while the sampling approach covers more than half of the national cow population, it may miss some positive animals in the farms. These groups may be underrepresented but are essential to illness persistence and transmission. Third, provincial differences in sampling intensity and reporting quality may explain heterogeneity in prevalence rather than epidemiological variation. Some provinces reported no positive isolates, possibly due to insufficient sampling volumes or inconsistent surveillance coverage. Finally, the study did not assess abortion‐related materials or farm‐ or management‐related risk factors, such as animal movement, biosecurity practices, vaccination history, or interspecies contact, which could have illuminated transmission pathways and the reasons for persistent infection despite vaccination.

## Author Contributions

Saeed Alamian and Maryam Dadar: writing–review and editing, writing–original draft, resources, project administration, methodology, investigation, formal analysis, and conceptualization. Karim Amiry and Akram Bahreinipour: writing–review and editing, resources, methodology, and conceptualization.

## Funding

This study was supported by the grant 3‐18‐18‐57‐006010331 from the Razi Vaccine and Serum Research Institute (RVSRI), Agricultural Research, Education and Extension Organization (AREEO), and by the Iran National Science Foundation (INFS) under project no. 99030922.

## Disclosure

All authors have read and approved the final manuscript.

## Conflicts of Interest

The authors declare no conflicts of interest.

## Supporting Information

Additional supporting information can be found online in the Supporting Information section.

## Supporting information


**Supporting Information** Supporting 1. Table S1. Annual cattle population, number of blood samples collected, and positive cases by province. Supporting 2. Table S2. Primer sets and expected amplicon sizes for different *Brucella* species.

## Data Availability

Data are available on reasonable request due to privacy/ethical restrictions.
