# Supplementary material for: Epidemiological Trends and Strain Distribution of Bovine Brucellosis in Vaccinated Industrial Dairy Cattle (2021–2024)
Source: Vet Med Int. 2026 May 15;2026:7432813. doi: 10.1155/vmi/7432813 (PMC13179448; doi:10.1155/vmi/7432813)
Supplement: Supplementary file 1 — Supporting Information Supporting 1. Table S1. Annual cattle population, number of blood samples collected, and positive cases by province. Supporting 2. Table S2. Primer sets and expected amplicon sizes for different Brucella species. [file VMI-2026-7432813-s001.docx]

**Supplementary Material**

**Table S1. Annual cattle population, number of blood samples collected, and positive cases by province**

| Provinces | Years | Total animals Farm (animal) | Blood sampling  Farm (animal) | Positive samples  Farm (animal) |
| --- | --- | --- | --- | --- |
| East Azerbaijan | 2021  2022  2023  2024 | 253 (39294)  264 (51317)  268 (48980)  274 (116544) | 253 (15851)  264 (21555)  268 (24988)  274 (62203) | 6 (10)  8 (18)  4 (12)  14 (122) |
| West Azerbaijan | 2021  2022  2023  2024 | 250 (27824)  231 (25885)  205 (26806)  174 (20783) | 250 (14651)  231 (13364)  205 (14622)  174 (10260) | 0 (0)  3 (4)  1 (1)  3 (8) |
| Ardabil | 2021  2022  2023  2024 | 61 (26587)  71 (43551)  42 (27648)  27 (28648) | 61 (22499)  71 (30155)  42 (24304)  27 (23971) | 11 (15)  10 (10)  2 (2)  3 (3) |
| Isfahan | 2021  2022  2023  2024 | 603 (574500)  626 (613287)  609 (518177)  592 (520735) | 603 (257483)  626 (297190)  609 (259309)  592 (268055) | 141 (754)  113 (331)  122 (453)  80 (316) |
| Alborz | 2021  2022  2023  2024 | 308 (299182)  305 (224055)  331 (236787)  298 (307752) | 308 (132268)  305 (98061)  331 (98237)  298 (131489) | 38 (154)  49 (150)  60 (160)  48 (197) |
| Ilam | 2021  2022  2023  2024 | 80 (5382)  109 (5390)  93 (5248)  98 (5766) | 80 (3375)  109 (3561)  93 (3397)  98 (3718) | 1 (1)  0 (0)  1 (2)  3 (3) |
| Bushehr | 2021  2022  2023  2024 | 27 (1559)  28 (1292)  20 (1412)  25 (2467) | 27 (564)  28 (547)  20 (788)  25 (716) | 1 (1)  1 (1)  1 (1)  2 (2) |
| Tehran | 2021  2022  2023  2024 | 1009 (816925)  1090 (890477)  1017 834442)  954 (664164) | 1009 (453030)  1090 (474834)  1017 (436673)  954 (354721) | 111 (3074)  163 (941)  117 (368)  89 (181) |
| Chaharmahal and Bakhtiari | 2021  2022  2023  2024 | 408 (35481)  434 (36456)  601 (74619)  824 (82347) | 408 (19300)  434 (24694)  601 (46582)  824(47740) | 17 (28)  34 (105)  50 (167)  99 (230) |
| South Khorasan | 2021  2022  2023  2024 | 240 (14788)  417(22972)  445 (23182)  403 (21216) | 240 (10358)  417 (15376)  445 (15763)  403 (14336) | 35 (49)  8 (8)  2 (6)  15 (25) |
| Khorasan Razavi | 2021  2022  2023  2024 | 683 (196151)  678 (204369)  760 (183703)  646 (173714) | 683 (90336)  678 (87028)  760 (87313)  646 (79681) | 41 (93)  40 (152)  19 (28)  15 (25) |
| North Khorasan | 2021  2022  2023  2024 | 58 (6476)  161 (14776)  115 (11941)  95 (11057) | 58 (3615)  161 (9544)  115 (8867)  95 (7811) | 2 (2)  2 (2)  1 (1)  2 (2) |
| Khuzestan | 2021  2022  2023  2024 | 109 (15118)  58 (18831)  54 (13895)  47 (12035) | 109 (6313)  58 (6828)  54 (6552)  47 (6797) | 0 (0)  1 (2)  1 (3)  3 (10) |
| Zanjan | 2021  2022  2023  2024 | 105 (27560)  105 (28929)  107 (29096)  111 (29268) | 105 (9697)  105 (12069)  107 (12716)  111 (12984) | 2 (2)  0 (0)  3 (7)  2 (3) |
| Semnan | 2021  2022  2023  2024 | 252 (31005)  296 (37025)  323 (35122)  260 (32597) | 252 (12705)  296 (19066)  323 (19169)  260 (18507) | 16 (41)  18 (30)  17 (44)  7 (9) |
| Sistan and Baluchestan | 2021  2022  2023  2024 | 26 (22138)  11 (8164)  6 (5254)  7 (3338) | 26 (2218)  11 (4401)  6 (1774)  7 (1021) | 1 (3)  0 (0)  0 (0)  0 (0) |
| Fars | 2021  2022  2023  2024 | 622 (102581)  693 (129750)  649 (157933)  564 (107461) | 622 (56696)  693 (62956)  649 (97598)  564 (64851) | 55 (356)  84 (292)  68 (437)  33 (122) |
| Qazvin | 2021  2022  2023  2024 | 357 (261748)  368 (341811)  322 (251414)  328(277979) | 357 (261778)  368 (153053)  322 (125994)  328 (141805) | 23 (124)  26 (46)  19 (68)  13 (22) |
| Qom | 2021  2022  2023  2024 | 236 (106776)  565 (102664)  635 (73644)  487 (54483) | 236 (25276)  565 (41172)  635 (38914)  487 (27966) | 64 (270)  140 (441)  98 (194)  16 (36) |
| Kurdistan | 2021  2022  2023  2024 | 176 (19008)  162 (18342)  154 (40923)  139 (29729) | 176 (9320)  162 (9371)  154 (22217)  139 (15745) | 1 (4)  0 (0)  5 (19)  4 (8) |
| Kerman | 2021  2022  2023  2024 | 522 (45853)  600 (58013)  603 (72822)  568 (62241) | 522 (27811)  600 (34518)  603 (34293)  568 (31803) | 18 (30)  20 (71)  22 (68)  20 (33) |
| South Kerman | 2021  2022  2023  2024 | 16 (563)  9 (477)  4 (110)  3 (85) | 16 (300)  9 (477)  4 (54)  3 (32) | 1 (1)  9 (161)  0 (0)  0 (0) |
| Kermanshah | 2021  2022  2023  2024 | 163 (72342)  163 (87260)  164 (48443)  148 (49888) | 163 (33941)  163 (50280)  164 (24365)  148 (22097) | 11 (113)  5 (14)  1 (3)  0 (0) |
| Kohgiluyeh and Boyer Ahmad | 2021  2022  2023  2024 | 79 (7508)  75 (7082)  74 (21442)  68 (16109) | 79 (3922)  75 (4440)  74 (11475)  68 (7641) | 3 (3)  3 (3)  7 (28)  3 (5) |
| Golestan | 2021  2022  2023  2024 | 177 (25004)  218 (31846)  192 (29586)  171 (31966) | 177 (9964)  218 (12262)  192 (10329)  171 (12823) | 2 (2)  8 (9)  12 (17)  5 (10) |
| Gilan | 2021  2022  2023  2024 | 32 (3035)  60 (8018)  55 (9017)  44 (4199) | 32 (1945)  60 (2591)  55 (2195)  44 (1880) | 0 (0)  1 (1)  0 (0)  1 (1) |
| Lorestan | 2021  2022  2023  2024 | 182 (20978)  367 (47926)  358 (36329)  370 (36225) | 182 (7869)  367 (18475)  358 (12138)  370 (12809) | 10 (148)  17 (101)  11 (23)  8 (15) |
| Mazandaran | 2021  2022  2023  2024 | 71 (13011)  130 (53587)  123 (41762)  96 (21965) | 71 (4499)  130 (15595)  123 (12618)  96 (13174) | 0 (0)  0 (0)  0 (0)  0 (0) |
| Markazi | 2021  2022  2023  2024 | 86 (67235)  125 (96417)  119 (79511)  125 (140874) | 86 (24018)  125 (50926)  119 (39002)  125 (66329) | 3 (1)  6 (92)  0 (0)  11 (21) |
| Hormozgan | 2021  2022  2023  2024 | 4 (1862)  0 (0)  2 (824)  0 (0) | 4 (455)  0 (0)  2 (412)  0 (0) | 0 (0)  0 (0)  0 (0)  0 (0) |
| Hamedan | 2021  2022  2023  2024 | 169 (48648)  164 (44664)  185 (65506)  171 (48274) | 169 (21276)  164 (21724)  185 (39186)  171 (30671) | 10 (75)  3 (3)  19 (87)  13 (176) |
| Yazd | 2021  2022  2023  2024 | 311 (53827)  340 (71075)  333 (62701)  311 (55018) | 311 (19373)  340 (23983)  333 (22806)  311 (19124) | 59 (218)  94 (543)  84 (256)  53 (146) |
| Total | 2021  2022  2023  2024 | 7675 (2989949)  8923 (3325708)  8968 (3068279)  8428 (2968927) | 7675(1562706)  8923 (1620096)  8968 (1546135)  8428 (1512760) | 683 (5572)  866 (3531)  747 (2455)  565 (1731) |

**Table S2. Primer sets and expected amplicon sizes for different *Brucella* species**

| Strain amplicon | Primer set | Primer sequence (5-3’) | DNA target | size (bp) | References |
| --- | --- | --- | --- | --- | --- |
| wboA gene | wboA gene | TTAAGCGCTGATGCCATTTCCTTCAC  TTTAGTTTGCCGTAATATAGGTCTAGAACCTGT  GCCAACCAACCCAAATGCTCACAA | wboA gene | 400 bp for field B. abortus 900 and 1,300 bp for RB51 DNA segment | [15] |
| AMOS PCR | IS711  AB | TGCCGATCACTTTCAAGGGCCTTCAT  GACGAACGGAATTTTTCCAATCCC | IS711 | 498 | [15] |
| AMOS PCR | IS711  BM | TGCCGATCACTTTCAAGGGCCTTCAT AAATCGCGTCCTTGCTGGTCTGA | IS711 | 731 | [15] |
| AMOS PCR | IS711  B.ovis | TGCCGATCACTTTCAAGGGCCTTCAT  CGGGTTCTGGCACCATCGTCG | IS711 | 976 | [15] |
| AMOS PCR | IS711  B.suis | TGCCGATCACTTTCAAGGGCCTTCAT  GCGCGGTTTTCTGAAGGTTCAGG | IS711 | 285 |  |
